# Supplementary material for: A Critical View of the Application of the APEX Software (Aqueous Photochemistry of Environmentally-Occurring Xenobiotics) to Predict Photoreaction Kinetics in Surface Freshwaters
Source: Molecules. 2019 Dec 18;25(1):9. doi: 10.3390/molecules25010009 (PMC7017383; doi:10.3390/molecules25010009)
Supplement: Supplementary file 1 [file molecules-25-00009-s001.zip › 3.2.4_gcc-4.4.0.zip!/3.2.4_gcc-4.4.0/3.2.4_gcc-4.4.0/doc/octave/html/interpreter/Famous-Matrices.html]

Famous Matrices - Untitled


Previous: Special Utility Matrices,
Up: Matrix Manipulation


---

### 16.5 Famous Matrices

The following functions return famous matrix forms.

— Function File: **hadamard** (n)  
> Construct a Hadamard matrix Hn of size n-by-n. The
> size n must be of the form `2 ^` k `*` p in which
> p is one of 1, 12, 20 or 28. The returned matrix is normalized,
> meaning `Hn(:,1) == 1` and `H(1,:) == 1`.
>
> Some of the properties of Hadamard matrices are:
>
> - `kron (`Hm`,` Hn`)` is a Hadamard matrix of size
>   m-by-n.- `Hn * Hn' ==` n `* eye (`n`)`.- The rows of Hn are orthogonal.- `det (`A`) <= abs(det (`Hn`))` for all A with
>         `abs (`A `(`i`,` j`)) <= 1`.- Multiply any row or column by -1 and still have a Hadamard matrix.

— Function File: **hankel** (c, r)  
> Return the Hankel matrix constructed given the first column c, and
> (optionally) the last row r. If the last element of c is
> not the same as the first element of r, the last element of
> c is used. If the second argument is omitted, it is assumed to
> be a vector of zeros with the same size as c.
>
> A Hankel matrix formed from an m-vector c, and an n-vector
> r, has the elements
>
> ```
>           H(i,j) = c(i+j-1),  i+j-1 <= m;
>           H(i,j) = r(i+j-m),  otherwise
> ```
>
>
>
>
> **See also:** vander, sylvester\_matrix, hilb, invhilb, toeplitz.

— Function File: **hilb** (n)  
> Return the Hilbert matrix of order n. The
> i, j
> element of a Hilbert matrix is defined as
>
> ```
>           H (i, j) = 1 / (i + j - 1)
> ```
>
>
>
>
> **See also:** hankel, vander, sylvester\_matrix, invhilb, toeplitz.

— Function File: **invhilb** (n)  
> Return the inverse of a Hilbert matrix of order n. This can be
> computed exactly using
>
> ```
>                       (i+j)         /n+i-1\  /n+j-1\   /i+j-2\ 2
>            A(i,j) = -1      (i+j-1)(       )(       ) (       )
>                                     \ n-j /  \ n-i /   \ i-2 /
>           
>                   = p(i) p(j) / (i+j-1)
> ```
>
> where
>
> ```
>                        k  /k+n-1\   /n\
>               p(k) = -1  (       ) (   )
>                           \ k-1 /   \k/
> ```
>
> The validity of this formula can easily be checked by expanding
> the binomial coefficients in both formulas as factorials. It can
> be derived more directly via the theory of Cauchy matrices:
> see J. W. Demmel, Applied Numerical Linear Algebra, page 92.
>
> Compare this with the numerical calculation of `inverse (hilb (n))`,
> which suffers from the ill-conditioning of the Hilbert matrix, and the
> finite precision of your computer's floating point arithmetic.
>
>
>
> **See also:** hankel, vander, sylvester\_matrix, hilb, toeplitz.

— Function File: **magic** (n)  
> Create an n-by-n magic square. Note that `magic
> (`2`)` is undefined since there is no 2-by-2 magic square.

— Function File: **pascal** (n, t)  
> Return the Pascal matrix of order n if t `= 0`.
> t defaults to 0. Return lower triangular Cholesky factor of
> the Pascal matrix if t `= 1`. This matrix is its own
> inverse, that is `pascal (`n`, 1) ^ 2 == eye (`n`)`.
> If t `= -1`, return its absolute value. This is the
> standard pascal triangle as a lower-triangular matrix.
> If t `= 2`, return a transposed and permuted version of
> `pascal (`n`, 1)`, which is the cube-root of the identity
> matrix. That is `pascal (`n`, 2) ^ 3 == eye (`n`)`.
>
>
>
> **See also:** hankel, vander, sylvester\_matrix, hilb, invhilb, toeplitz, hadamard, wilkinson, compan, rosser.

— Function File: **rosser** ()  
> Returns the Rosser matrix. This is a difficult test case used to test
> eigenvalue algorithms.
>
>
>
> **See also:** hankel, vander, sylvester\_matrix, hilb, invhilb, toeplitz, hadamard, wilkinson, compan, pascal.

— Function File: **sylvester\_matrix** (k)  
> Return the Sylvester matrix of order
> n = 2^k.
>
>
>
> **See also:** hankel, vander, hilb, invhilb, toeplitz.

— Function File: **toeplitz** (c, r)  
> Return the Toeplitz matrix constructed given the first column c,
> and (optionally) the first row r. If the first element of c
> is not the same as the first element of r, the first element of
> c is used. If the second argument is omitted, the first row is
> taken to be the same as the first column.
>
> A square Toeplitz matrix has the form:
>
> ```
>           c(0)  r(1)   r(2)  ...  r(n)
>           c(1)  c(0)   r(1)  ... r(n-1)
>           c(2)  c(1)   c(0)  ... r(n-2)
>            .     ,      ,   .      .
>            .     ,      ,     .    .
>            .     ,      ,       .  .
>           c(n) c(n-1) c(n-2) ...  c(0)
> ```
>
>
>
>
> **See also:** hankel, vander, sylvester\_matrix, hilb, invhilb.

— Function File: **vander** (c, n)  
> Return the Vandermonde matrix whose next to last column is c.
> If n is specified, it determines the number of columns;
> otherwise, n is taken to be equal to the length of c.
>
> A Vandermonde matrix has the form:
>
> ```
>           c(1)^(n-1) ... c(1)^2  c(1)  1
>           c(2)^(n-1) ... c(2)^2  c(2)  1
>               .     .      .      .    .
>               .       .    .      .    .
>               .         .  .      .    .
>           c(n)^(n-1) ... c(n)^2  c(n)  1
> ```
>
>
>
>
> **See also:** hankel, sylvester\_matrix, hilb, invhilb, toeplitz.

— Function File: **wilkinson** (n)  
> Return the Wilkinson matrix of order n.
>
>
>
> **See also:** hankel, vander, sylvester\_matrix, hilb, invhilb, toeplitz, hadamard, rosser, compan, pascal.
